# Knowledge and skills needed to improve as preceptor: development of a continuous professional development course – a qualitative study part I

**DOI:** 10.1186/s12912-015-0103-9

**Published:** 2015-10-16

**Authors:** Mariette Bengtsson, Elisabeth Carlson

**Affiliations:** Faculty of Health and Society, Department of Care Science, Malmö University Sweden SE, 20506 Malmö, Sweden

**Keywords:** Advanced level, Clinical practice, Continuous professional development course, Development, Preceptor

## Abstract

**Background:**

Preceptors are expected to have the skills to be able to form an effective learning environment and facilitate a constructive clinical learning experience for students and new employees. Internationally, access to education for preceptors varies, with preceptors worldwide requesting more education in preceptorship. This article is based on a two-part study focusing on both the development and evaluation of a continuous, credit-bearing professional development course. The aim of this part of the study was to investigate and include preceptors’ requests and educational needs when developing a continuous professional development course on an advanced level.

**Methods:**

This study used a qualitative research approach. In total, 64 preceptors (62 women and two men) answered one single written, self-administered global question online. The participants were all interested in teaching and had completed an undergraduate training in preceptorship. The collected data was analysed by content analysis inspired by Burnard’s description of the method.

**Results:**

The participating preceptors illuminated two main themes: ‘Tools for effective precepting of students and healthcare professionals’ and ‘in-depth knowledge and understanding of preceptorship in an academic setting’. The results suggest that vital components for preceptor preparation could be a) teaching and learning strategies, b) reflective and critical reasoning, c) communication models, d) the role of the preceptor, and e) preceptorship.

**Conclusion:**

Using the results from this study as a guide, a continuous professional development course was designed to assist preceptors in deepening their knowledge of preceptorship in regard to planning, leading and implementing educational activities directed at students, healthcare professionals, patients and their families. The course content focuses on skills needed for preceptorship and is based on adult learning principles. A continuous, credit-bearing professional development course must include an exam by which participants are formally assessed and graded; therefore, a written assignment was included as part of the course.

## Background

Clinical practice is agreed to be an essential part of all healthcare professions in which preceptors play an important role [1, 3]. According to the Bologna Process in Europe, clinical education should be incorporated into all courses in Bachelor of Nursing programmes and constitute half of the programme [4]. Over a certain time period, established healthcare professionals guide and direct undergraduate and graduate students as well as newly educated colleagues and play an invaluable role in their clinical learning and training [1, 5, 6]. Newcomers must become immersed in new clinical settings and be able to learn their profession and further develop as healthcare professionals [7]. They need to socialise in this environment and be given an opportunity to practice the knowledge and skills taught at the campus [6, 8]. Guided by an experienced preceptor, newcomers are required to integrate cognitive, affective and psychomotor skills [9]. However, learning the art and science of a healthcare profession is a complex task.

Preceptorship is about teaching ethical dimensions, uniting theory and praxis, and is a complex and dynamic educational process which comprises the design and implementation of various learning strategies, evaluation, and assessment [10, 11]. The scope of the preceptor’s role is diverse; for example, coaching, guiding, inspiring, teaching and role-modelling with intrinsic rewards and extrinsic demands [1, 2]. The relationship between the preceptor and a student is fundamental in clinical practice and should be grounded in mutual openness in a supportive manner [12]. Preceptors are expected to offer their adepts the best conditions possible to allow them to develop in their profession [8]. Preceptors are expected to create an effective learning environment and facilitate a constructive clinical learning experience. However, simply assigning a newcomer to a named preceptor is no guarantee for the quality of the clinical training. If someone is trained by a non-supportive, unskilled preceptor and thus not gaining confidence, feedback, and guidance, the newcomer’s learning may be less than effective [13, 14].

The traditional assumption is that healthcare professionals enter their profession naturally inclined to precept and inherently possess the needed skills for this assignment. They are expected to have the skills required to know how and when to share their knowledge and to have the capacity to know when to give newcomers increased independence. Students operate under a registered nurse license, and a major concern among preceptors is when students demonstrate inconsistency between their reasoning and their actions, thus performing unsafe care [15]. In these situations, preceptors want to discuss their concerns and be supported by an experienced colleague. The increased stress that comes with the teaching responsibilities of difficult situations takes the vigour out of the preceptors, causing exhaustion and stress [16]. Therefore, it is important to give preceptors assistance when needed so they will continue to be preceptors in the future despite having had a tough experience. Thus, it cannot be assumed that healthcare professionals can automatically function as a preceptor in a stressful and complex healthcare environment without any pedagogical knowledge, practice and support [3, 7]. Preceptors are required to possess professional as well as academic qualifications, and employers have a responsibility to provide preceptors with the knowledge and skills required to provide preceptorship [5]. However, the employers do not always fulfil these requirements. Access to education for preceptors varies both nationally and internationally, along with the extent, content, and demands of the existing courses [17, 18]. In general, international healthcare professionals request more education in preceptorship [2, 7]. Preceptors have also indicated that courses for preceptors do not always provide the content that the preceptors request [19]. Therefore, it is important to pay attention to the preceptors’ needs before developing a continuous, credit-bearing professional development (CPD) course. The aim of this study was to investigate and include preceptors’ requests and educational needs when developing a CPD course on an advanced level.

## Methods

This study used a qualitative research approach, using one single written, self-administered global question, analysed by content analysis which was inspired by Burnard’s description of the method [20–22].

### Participants and baseline assessments

In the current study, a purposive sampling was used. The participants were preceptors working in the healthcare sector in south of Sweden who had a special interest in clinical education. They had all completed undergraduate training in preceptorship corresponding to 7.5 ECTS credits, and at the completion of the course, the participants were informed about this forthcoming study. In autumn 2011 the participants were invited to take part in this study. In total, 64 preceptors (62 women and two men) were invited, of which all volunteered and were thus included. All of the participants were prepared for their preceptorship, teaching, and assessment, but wanted to expand their knowledge and skills to be able to further develop as a preceptor. The mean age of the preceptors was 43 years, ranging from 25–63 years, and they had been working as preceptors in a clinical setting from between 5–20 years by this time.

### Study design

Online, the participants answered the study question: “What further knowledge and skills do you need to develop as a preceptor?” Usually, the best way to gather persons’ perceptions is by extensive verbal interviews, but qualitative researchers must always consider their pre-understanding of the research area and attempt to minimise any bias from their preconceived knowledge. Both authors (EC, MB) are teachers involved in the supervision or in the education programme of preceptors. Therefore, it was acknowledged that there was a risk that the authors’ own perceptions (deliberate or accidental) could influence the preceptors’ perspectives in an interview situation, thus placing bias on the interpretation of the data. As a result, a written question was used. The use of only one written question may appear to be insufficient, but according to previous studies [23–25] a single question can provide information on all aspects of a phenomenon and is sufficient to summarise any given individual’s perceptions. The total amount of text based on the preceptors’ reflections concerning the study question totalled 64 pages with 12,116 words which ranged between 106 and 456 words per each preceptor (mean = 224).

### Data analysis

Content analysis, which can be used on all textual data [21], was chosen for this study. By using content analysis, the researcher explores textual data with the view to group together similar types of statements and to construct a systematic list of themes and categories. For the current study, the concept of Burnard’s method for content analysis was chosen [20–22]. The authors (EC, MB) started the analysis by reading all 64 pages of text several times to become familiar with the data. Key issues, so-called *meaning units*, were coded, grouped and listed. To increase validity, the two authors analysed the text separately and then compared and discussed their listed categories and subheadings. The outcome of the discussion was a slightly adjusted new list. After establishing a form of consensus, the original text was analysed and coded again to ensure that the categories and subheadings in the final list covered all aspects of the participants’ answers.

### Ethical aspects

The study was conducted in accordance with the Helsinki Declaration [26], and ethical approval was obtained by the Ethical Advisory Board at the university (HS 60-11/919:2). All participants were informed about the study orally, as well as in writing, and were guaranteed confidentiality. They were informed that answering the study question was voluntary and that they could withdraw their data from the study at any time without this affecting their relationship with the university or the researchers. The participants gave written, informed consent before signing up for participation.

## Results

The overarching themes of ‘tools for effective precepting of students and healthcare professionals’ and ‘in-depth knowledge and understanding of preceptorship in an academic setting’ are the results of the preceptors’ suggestions for content in an education programme for preceptors. The preceptors considered the supervision of students to be difficult, at times burdensome and stressful. The preceptors wanted the concrete tools and teaching strategies to handle this pressure. The preceptors needed further knowledge and understanding of their role as a preceptor and educator and what responsibilities they may have. They wanted to be inspired to use new methods with the purpose of being flexible in the preceptorship and to be able to supervise even the most challenging students.

One critical point emphasised by the preceptors was their responsibility for the ongoing evaluation of the students or new graduates’ progress towards expected goals. The preceptors described their educational role as challenging, especially supervising students with weak theoretical knowledge or students who will not acknowledge their own weaknesses. They mentioned that the students are adults, and some of the students also have vast life experience which affects the preceptorship. To precept various groups of people is expected in all clinical settings, but the preceptors felt that the expectations on preceptors needs to be clarified. The identified themes, categories and subheadings are presented in Table 1 and further discussed later on.

**Table 1:** Overview of identified themes, categories, and subheadings based on the preceptors’ perspectives

*Theme*	
Tools for effective precept of students and healthcare professionals	
*Categories*	*Subheadings*
Knowledge about activities, clinical teaching and learning strategies	Teaching and learning strategies
	Concrete tools
	Adult learning principles
	Principles for rational assessment
Knowledge and skills about reflective and critical reasoning.	Reflection in clinical practice
	Self-assessment
Knowledge and skills about communication models	Communication skills
	Principles for communication
	Constructive criticism
*Theme*	
In-depth knowledge and understanding of preceptorship in an academic setting	
*Categories*	*Subheadings*
How to develop as a preceptor	Support, coaching and training Competence in a life-long perspective
To precept in a scientific perspectives	Teaching and learning models Science of education

### Tools for effective precepting of students and healthcare professionals

This theme focuses on the practical implications of preceptorship, including the three categories: ‘Knowledge about activities, clinical teaching and learning strategies’, ‘Knowledge and skills about reflective and critical reasoning’, and ‘Knowledge and skills about communication models’. The preceptors expressed the need for more knowledge about teaching and learning strategies and also about adult learning principles in order to develop their preceptorship. They also requested concrete teaching tools, advice, and methods to use in their clinical teaching. The importance of communication strategies to use in difficult situations was also highlighted.

### Knowledge about activities, clinical teaching and learning strategies

The preceptors wanted to learn more about **teaching and learning strategies** to ensure they can demonstrate the components students need to achieve their identified learning needs and goals. They requested new ideas and recommendations to better facilitate the adult students’ teaching, learning and assessment and to create a safe and meaningful experience for the students at the clinic:

*“When planning and preparing prior to the students starting their clinical practice, I need to be prepared for teaching situations to feel more confident and to have structure from the beginning.”* P 32

The preceptors wanted the more concrete tools which comes from further knowledge and the skills for how to incorporate the theoretical understanding of preceptorship into practice when mentoring new preceptor colleagues. They wanted to know how to modify and match their own teaching methods into their colleagues teaching and learning styles. Concrete tools were requested to facilitate discussions with new preceptors when difficult dilemmas arise:

*“…tools for improved guidance and arguments to critically discuss dilemmas that new preceptors at the clinic describe, for example, to determine whether appropriate levels of education are upheld or how to discuss a student’s personality.*” P 12

The preceptors wanted to gain knowledge about **adult learning principles** and new ideas of how to best practice preceptorship in an interprofessional group of students. Each student needs to reach the capability to see beyond the context, and to understand and develop their own profession:

*“I need to know more about adult learning principles to improve my teaching strategies related to profession-specific and inter-professional preceptorship.”* P 38

The preceptors also wanted more knowledge about **rational assessment principles**. They need to be autonomous when assessing students to achieve a rational assessment. Their assessment must be based on the objective judgement of the student so that the preceptors’ personal opinions do not influence the assessment:

*“….identify resources to supervise and assess students in a respectable and fair manner, even if the personal chemistry between the student and the preceptor do not match … personal perceptions should not influence the actual assessment of the students’ performance.”* P 16

### Knowledge and skills about reflective and critical reasoning

**Reflection in clinical practice** was highlighted by the preceptors. They wanted to know how to increase their ability to facilitate learning among newcomers made up of students as well as new graduates by encouraging reflection on patient care and organising a variety of reflective activities.

*“…more about how I, as a preceptor, can use reflection in different contexts and settings in which both I and the person in training can feel comfortable and develop.”* P 27

In addition, the preceptors requested more knowledge about **self-assessment**. They wanted to help students to better critique and realistically analyse their own performance. The preceptors mentioned that students do not always express realistic goals for their clinical training. Guidance on how to help the students focus on what is relevant so the students can track their progress was requested:

*“…learn more about self-assessment in a way that can lead the students’ progress in their learning and professional development.”* P 10

### Knowledge and skills about communication models

The preceptors wanted further training to develop their **communication skills**, because they also take part in difficult discussions about students’ educational needs with colleagues and teachers from the university, as well as with the students.

*“I want to improve my ability to discuss with students, teachers and colleagues when planning and guiding clinical training as well as when assessing student performance.”* P 33

Knowledge about the **principles for communication** was also requested. The preceptors mentioned that difficult situations and discussions occur during the students’ clinical practice; for example, when there is the risk of failing the student. The preceptors wanted to improve their ability to communicate with students during these circumstances. They felt misunderstood, and their comments were sometimes taken badly by the students. The preceptors perceive these discussions as stressful for both themselves and the students, and they want to avoid these discussions as much as possible:

*“I need further knowledge of conversational methods to manage problematic discussions and to be able to keep the conversation focused and structured.”* P 46

The preceptors highlighted that giving and receiving **constructive criticism** is a big issue*.* They felt concern for the students as individuals in situations where they do not meet the expectations of the educational or the clinical setting. The preceptors mention the difficulty of critiquing in a positive manner and had experienced a loss of self-reliance in some students. They requested tools that could strengthen students’ self-confidence in these situations:

*“…more knowledge about giving and receiving constructive criticism without affecting the students' self-confidence.”* P 57

### In-depth knowledge and understanding of preceptorship in an academic setting

The preceptors feel that the expectations on a preceptor needs clarification, and they wanted to increase their position of being a preceptor. This theme focuses on two aspects; namely, How to develop as a preceptor and **preceptorship in a scientific perspective**.

### How to develop as a preceptor

The preceptors in this study wanted to develop and feel a sense of security in their preceptor role, especially given that it is regarded as a demanding responsibility. They thought critical friends could be of help, giving **support, coaching, and training** to verbalise their own unique teaching style. The preceptors wanted to receive feedback on their role as a preceptor.

*“I want to be evaluated as a preceptor to gain insight into what I need to improve, and therefore, I need critical friends.”* P 22

The preceptors wanted to know more about how they could learn and how to use that knowledge in their preceptorship. Someone had heard about portfolios and thought that this concept may be useful in gaining a deeper understanding of their own **competence from a lifelong perspective**, but wanted more knowledge before implementing it:

*“I need to become aware of my own learning style and how to develop as a tutor in relation to lifelong learning.”* P 64

### To precept from a scientific perspective

The preceptors wanted enhanced pedagogical perspectives on **teaching and learning models** and how to implement new teaching methods. They perceived a lack of depth and breadth in their theoretical teaching knowledge, leaving them insufficient in sorting out arguments for competent preceptorship:

*“…be able to critically examine various educational theories as well as teaching and learning models and argue for its importance in clinical training settings.”* P 2

The preceptors wanted insights and some understanding of the academic world as well as more knowledge about the **science of education**. They wanted to be comfortable in using science and research, and thus be able to discuss precepting from a scientific perspective:

*“I need to become more familiar with research regarding preceptorship to be able to gain in-depth knowledge and understanding of teaching and learning strategies, to be able to deepen my discussions with colleagues.”* P 41

## Discussion

To our knowledge, this is the first study where the requests and needs of preceptors guided the development of a CPD course on an advanced level. Baltimore [5] argues that this approach to course development is the best starting point. The preceptors requested knowledge and understanding of preceptorship in two areas, namely, the concrete practical level and the theoretical, scientific level. This falls in line with the literature discussing nursing preceptor programs [17, 27]. The preceptors in previous studies requested teaching strategies and concrete tools to better precept students during their clinical placement. Although the preceptors that took part in the current study already had pedagogical education on undergraduate level, they argued for further education in order to improve their teaching. They asked specifically for adult learning principles placed in a scientific perspective. Precepting students today is a collaborative process between academia and practice. Altmann [27] and Eddy [17] agree with this view, and by practicing adult teaching principles, the preceptors are more likely to facilitate their own readiness to use the same methods in their own teaching. Therefore, we recommend that didactical methods in a CPD course are based on student-active learning activities, for example, case scenarios and experience-based discussions. In this way, theory and practice, and knowledge and skills can interact and be optimally processed [5]. In addition, according to Baltimore [5], a CPD course requires structure and to be well organised, given that adult learners have little tolerance for disorganisation. The preceptors also claimed the need for some understanding of how to teach students when precepting does not work. Baltimore [5], and Hilli and associates [11] claimed that preceptors first need to learn how to identify each learner’s essential needs and weaknesses to ensure that the preceptors are offering what the learner needs before using new teaching and learning strategies. From this perspective, it is crucial that a trusting relationship is established between preceptor and student despite the power differences between them [10, 12]. Further, the preceptors stated that feedback could be badly given and badly taken, thus concrete tools for clarifying critique were requested. Baltimore [5] agrees that providing constructive and helpful feedback can be challenging for preceptors, but one of the characteristics of an effective preceptor includes good communication skills.

The preceptors also wanted to develop their ability to strengthen students’ critical and reflective reasoning. Critical reflections include both intuition and logic, and may be the key to resolving issues and complex situations [28]. A systematic literature review performed by Whitehead and associates [7] concluded that newly graduated nurses benefit from reflection and critical reasoning in action. However, preceptors can enable reflection by various techniques depending on the situation. By becoming conscious of their own way to reflect and analyse various situations and issues, it could be easier for preceptors to teach others. Green and colleagues [29] indicate that the use of a portfolio can facilitate reflection, but also provide evidence of knowledge, skills and experience, thus supporting the process of lifelong learning.

Adults learn best by integrating their own experience with new knowledge, for example, through discussions with peers [30, 31]. Learning is not only a cognitive process, but also a social, cultural and emotional process [6, 7]. According to MacDowell and associates [32], preceptors also need to integrate and socialise with those from other professions to acquire interprofessional knowledge and skills which could be useful in their own preceptorship. Based on the findings in our study, we suggest that CPD courses should be open to all healthcare professions and that this approach could create positive attitudes among the participants which could then be applied in their preceptorship.

Some participants in the present study mentioned that they were perceived as “experts in in the field” by their colleagues, and wanted more in-depth insight in to how to support a colleague. They also wanted to strengthen the view of their performance as a preceptor. One way for preceptors to receive and train to provide support and feedback could be through a critical friend. A critical friend is a colleague who evaluates another colleague’s supervision through observation and by asking provocative questions [33, 34]. The critical friendship is based on a non-hierarchal relationship, which could be arranged in a CPD course where the participants could observe each other and then practice how to give feedback.

### Limitations

Given that participants only consisted of preceptors working in healthcare in south of Sweden and had a special interest in clinical teaching, the findings may not be generalisable to other settings. The most common way to collect qualitative data is by conducting interviews. However, we chose to collect data using one single written, self-administered question. As a result, we could obtain information from more preceptors, and this gave us a wider variety of educational needs to consider, but it also resulted in a deficiency in the depth of the answers.

## Conclusions

Based on the preceptors’ requests, a CPD course of 7.5 ECTS credits on an advanced level was developed. The CPD course was designed to assist preceptors in deepening their knowledge in preceptorship to be able to plan, lead and implement educational activities in all settings. Vital components of preceptor preparation in this course are a) teaching and learning strategies, b) communication skills, c) reflective and critical thinking, d) the role of preceptor, and e) preceptorship – all of which are based on the science of education.

Adult learning principly forms the approach, and a portfolio is used to highlight the development of the participants. At the end of the course, the preceptors are assessed and graded by a written assignment. After implementation of the course, the structure and content are to be evaluated to determine if the course fulfils the preceptors’ needs.

## Abbreviations

CPD: Continuous professional development
EC: Elisabeth Carlson
MB: Mariette Bengtsson
